# Efficacy of treatment based on TKIs in combination with PD-1 inhibitors for unresectable recurrent hepatocellular carcinoma

**DOI:** 10.1186/s12957-023-02939-5

**Published:** 2023-02-18

**Authors:** Ze Zhang, Tianyu Jiao, Junfeng Li, Bingyang Hu, Wenwen Zhang, Zhijun Wang, Tao Wan, Yafei Wang, Shichun Lu

**Affiliations:** 1 Medical School of Chinese People’s Liberation Army (PLA), Beijing, China; 2 Faculty of Hepato-Pancreato-Biliary Surgery, Chinese PLA General Hospital, Beijing, China; 3 Institute of Hepatobiliary Surgery of Chinese PLA, Beijing, China; 4 Key Laboratory of Digital Hepatobiliary Surgery, PLA, Beijing, China; 5 Department of Interventional Radiology, The First Medical Center, Chinese PLA General Hospital, Beijing, China

**Keywords:** Hepatocellular carcinoma, Recurrence, Tyrosine kinase inhibitors, PD-1 inhibitors

## Abstract

**Background and objective:**

The recurrence occurs within 5 years in up to 70% of hepatocellular carcinoma (HCC) patients who received radical liver resection, and most patients are no longer suitable for repeat surgery. There are limited treatment options for unresectable recurrent HCC. This study aimed to explore the potential efficacy of treatment based on TKIs in combination with PD-1 inhibitors for unresectable recurrent HCC.

**Methods:**

Forty-four patients with unresectable recurrent HCC after radical surgery between January 2017 and November 2022 were retrospectively collected and screened. All patients received the combination therapy of tyrosine kinase inhibitors (TKIs) and programmed cell death protein 1 (PD-1) inhibitors, and 18 of these patients received trans-arterial chemoembolization (TACE) or TACE combined with radiofrequency ablation (RFA). Two patients who received TKIs in combination with PD-1 inhibitors eventually obtained repeat surgery, with one patient undergoing a repeat hepatectomy and one patient receiving a liver transplant.

**Results:**

The median survival for these patients was 27.0 months (95% confidence interval [CI] 21.2, 32.8), with a 1-year overall survival (OS) rate of 83.6% (95% CI 77.9%, 89.3%). Median progression-free survival (PFS) was 15.0 months (95.0% CI 12.1, 17.9), with a 1-year PFS rate of 77.0% (95% CI 70.6%, 83.4%). The two patients who underwent repeat surgery had a survival time of 34 and 37 months after the combined treatment with no recurrence, respectively, as of November 2022.

**Conclusion:**

The combination of TKIs and PD-1 inhibitors for unresectable recurrent HCC is effective and can prolong the survival of patients in this group.

## Introduction

Hepatocellular carcinoma (HCC) is the sixth most common malignant neoplasm and the third leading cause of cancer deaths [1]. Liver resection remains the preferred treatment for patients with early-stage (Barcelona Clinic Liver Cancer, BCLC stage 0 or A) HCC [2]. However, tumor recurrence occurs in up to 70% of these patients within 5 years [3], and a majority of recurrent HCC patients were no longer suitable for repeat liver resection, such as multiple intrahepatic lesions, macrovascular invasion, or extrahepatic metastases [4].

Conventional treatment options for intrahepatic recurrence are varied and include repeat hepatectomy, trans-arterial chemoembolization (TACE), radiofrequency ablation (RFA), and liver transplantation [4, 5]. Patients with HCC who present with extrahepatic recurrent lesions are treated primarily with systemic therapy with or without loco-regional treatments of the intrahepatic lesions [6, 7]. Some studies have shown that repeat surgery can prolong the survival of selected patients with recurrent HCC [8–12]. However, there is a paucity of data on unresectable recurrent HCC.

Recently, anti-programmed death-ligand 1 (PD-L1) combined with anti-vascular endothelial growth factor (VEGF) has significantly prolonged the survival of patients with unresectable HCC [13]. In addition, based on similar principles, TKIs in combination with PD-1 inhibitors significantly prolonged the survival of patients with advanced HCC and increased the objective response rate (ORR) [14]. The latest guideline for the treatment of advanced HCC also recommends this strategy as a first-line treatment [15].

This study aimed to investigate the long-term prognosis of patients with unresectable recurrent HCC who received TKIs in combination with PD-1 inhibitors as the basic treatment.

## Materials and methods

### Patients

**Fig. 1:**
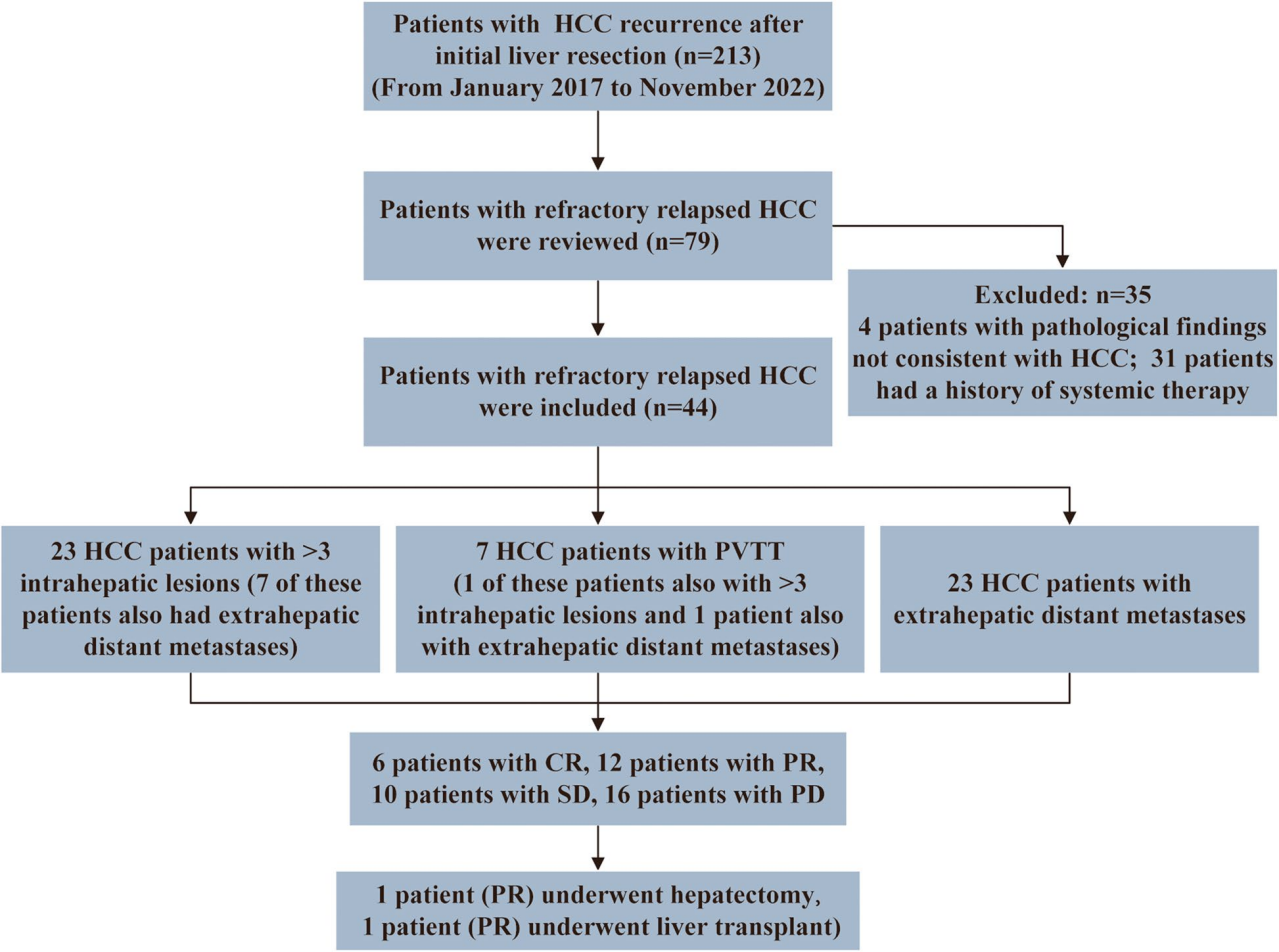
Flow diagram of the identification and selection of patients with HCC in this study (HCC, hepatocellular carcinoma; PVTT, portal vein tumor thrombosis; CR, complete response; PR, partial response; SD, stable disease; PD, progressive disease)

This study retrospectively collected 213 consecutive patients who were found to have HCC recurrence after initial hepatectomy of primary HCC from January 2017 to November 2022 at the Chinese People’s Liberation Army (PLA) General Hospital. Recurrence was diagnosed via pathological examination findings or with the non-invasive criteria used by the American Association for the Study of Liver Diseases [16]. Among these patients, 79 were diagnosed with unresectable recurrent HCC. We excluded 35 patients because they had a history of systemic therapy or histopathological findings not consistent with HCC (Fig. 1). The inclusion criteria for this study included (1) the recurrence of primary HCC after radical surgery; (2) upper abdominal magnetic resonance imaging (MRI) findings suggest at least one of the following features: (a) more than three recurrent lesions in the liver; (b) tumor invading macrovascular such as the portal vein, hepatic vein or inferior vena cava; and (c) extrahepatic metastases; (3) expected survival ≥ 12 weeks; (4) pathological examination confirms the diagnosis of HCC; (5) Eastern Cooperative Oncology Group performance status (ECOG PS) score ≤ 1; and (6) Child-Pugh score < 10. Exclusion criteria include (1) systemic therapy (including new molecular targeted drugs or immunotherapy drugs) before HCC recurrence and (2) history of hepatic encephalopathy and oesophageal or gastric variceal bleeding. The determination of the HCC tumor stage using the Barcelona Clinical Liver Cancer (BCLC) system and assessment of portal vein tumor thrombosis (PVTT) according to Japan’s liver cancer study group [17, 18]. PVTT can be graded as Vp1 (portal invasion at the third or more peripheral portal branch), Vp2 (portal invasion at the second portal branch), Vp3 (portal venous invasion at the first portal branch), and Vp4 (portal invasion at the main portal trunk) [17]. The study was approved by the ethics committee of the Chinese PLA General Hospital (Approval No. S2018-111-01).

### The treatment procedures

All patients received TKIs in combination with anti-PD-1 antibodies. TACE or RFA treatment depends on the size or the number of tumors and the anatomical location of the patient’s intrahepatic lesions. Lenvatinib (bodyweight ≥ 60 kg, 12 mg/day; < 60 kg, 8 mg/day) [14] or apatinib (750 mg/day) [19] were administered orally as TKIs. Anti-PD-1 antibodies were intravenously administered as follows: nivolumab (240 mg every 2 weeks) or pembrolizumab (200 mg every 3 weeks), or sintilimab [20] (200 mg every 3 weeks). TACE was performed by super-selective catheterization of the tumor-feeding hepatic arteries with the 2.7 F microcatheter (Progreat, Terumo Corporation, Japan). An emulsion of 6–20 mL of lipiodol (Lipiodol Ultrafluide; Guerbet, Paris, France), 30–50 mg of epirubicin (Farmorubicin; Pharmacia, Tokyo, Japan), 100–150 mg of oxaliplatin, 500–750 mg of 5-fluorouracil, 200–300 mg of calcium folinate, and 10–14 mg of mitomycin C (Kyowa Hakko Kogyo, Tokyo, Japan) were injected into the tumor supply vessels through the microcatheter. TKIs and Anti-PD-1 antibodies were initialized simultaneously, followed by TACE within 2 weeks. TKIs and anti-PD-1 antibodies were administered continuously until unacceptable toxicity or disease progression.

### Follow-up, outcome, and safety assessments

All patients are followed up every 2 months after the start of treatment. Blood tests include liver function, thyroid function, and tumor biomarkers. MRI was performed every 8–10 weeks, and Positron emission tomography-computed tomography (PET-CT) was performed as appropriate. Treatment outcomes were assessed for PFS, OS, ORR, disease control rate (DCR), and adverse events calculating PFS from the first day of combination therapy to tumor progression or death and OS from the first day of combination therapy to patient death. ORR is defined as the percentage of patients achieving complete response (CR) and partial response (PR). DCR is defined as the percentage of patients achieving CR, PR, and stable disease (SD). The last follow-up was performed on November 2022. Efficacy assessment is mainly based on modified response evaluation criteria in solid tumors (mRECIST) [21], and treatment-related adverse events were defined using the National Cancer Institute–Common Terminology Criteria for Adverse Events (NCI-CTCAE) version 5.0.

### Statistical analysis

We used SPSS 25.0 (SPSS, IL, USA) and R version 4.1.0 (Institute for Statistics and Mathematics, Vienna, Austria) to analyze and visualize our results. Quantitative data that conforms to a normal distribution were expressed as the means ± standard deviation. Non-normally distributed quantitative data were expressed as median values (range). Categorical data were expressed as a number (percentage). The PFS and OS were illustrated using Kaplan–Meier curves.

## Results

### Patient characteristics

A total of 44 patients were enrolled in this study (Table 1). The median duration of follow-up was 22 months (range, 5–54 months). Forty of the 44 patients were male (90.9%) with a mean age of 57 ± 8.9 years, and a mean body mass index (BMI) of 25.0 ± 3.3. 37 patients previously had hepatitis B infection (90.0%), 34 of whom had liver cirrhosis. Five patients (11.4%) had previous hepatitis C infections. Twenty-four patients (54.5%) recurred within 1 year after radical hepatectomy, while 20 patients (45.5%) recurred more than 1 year after radical hepatectomy. There were 23 patients with more than three intrahepatic lesions, seven with PVTT, 23 patients with extrahepatic metastases, seven patients with both more than three intrahepatic lesions and extrahepatic metastases, one patient with both more than three intrahepatic lesions and PVTT, and one patient with both extrahepatic metastases and PVTT in the study. Bone metastases, lung metastases, lymph node metastases, abdominal metastases, and diaphragmatic metastases were found in five, seven, three, five, and three patients, respectively. The liver function of most patients (90.9%) was normal (Child-Pugh A class). Furthermore, the mean levels of alanine aminotransferase, aspartate aminotransferase, albumin, and total bilirubin were 32.1 ± 23.6 U/L, 36.6 ± 23.1 U/L, and 41.0 ± 5.8 g/L, and 15.0 ± 6.9 mg/dL, respectively.

**Table 1:** Baseline characteristics of enrolled patients with HCC

Variable	Value, ***n***=44 (%)
Age (year)	57 ± 8.9
Sex male/female	40 (90.9%)/4 (9.1%)
BMI	25.0 ± 3.3
Drinking no/yes	15 (34.1%)/29 (65.9%)
HBsAg negative/positive	7 (15.9%)/37 (84.1%)
HCV negative/positive	39 (88.6%)/5 (11.4%)
Time interval from radical (hepatectomy to advanced recurrence) ≤ 1 year/> 1 year	24 (54.5%)/20 (45.5%)
Liver cirrhosis no/yes	10 (22.7%)/34 (77.3%)
Number of intrahepatic lesions ≤ 3/>3	21 (47.7%)/23 (52.3%)
PVTT classification	
No portal vein tumor thrombosis	37 (84.1%)
VP2	1 (2.3%)
VP3	4 (9.1%)
VP4	2 (4.5%)
Extrahepatic metastases	
No	21 (47.7%)
Yes	23 (52.3%)
Bone metastases	5 (11.4%)
Lung metastases	7 (15.9%)
Lymph node metastases	3 (6.8%)
Abdominal metastases	5 (11.4%)
Diaphragmatic metastases	3 (6.8%)
BCLC staging at recurrence B/C	15 (34.1%)/29 (65.9%)
Ascites no/yes	42 (95.5%)/2 (4.5%)
AFP (≤ 400 ng/mL)	38 (86.4%)
Child-Pugh classification A/B	40 (90.9%)/4 (9.1%)
ECOG performance 0/1	35 (79.5%)/9 (20.5%)
ALT (U/L)	32.1 ± 23.6
AST (U/L)	36.6 ± 23.1
ALB (g/L)	41.0 ± 5.8
Total bilirubin level (mg/dL)	15.0 ± 6.9

*BMI* body mass index, *HBsAg* hepatitis B surface antigen, *HCV* hepatitis C virus, *PVTT* portal vein tumour thrombosis, *BCLC* Barcelona Clinic Liver Cancer Group, *AFP* alpha-fetoprotein, *ECOG* Eastern Cooperative Oncology Group, *ALT* alanine aminotransferase, *AST* aspartate aminotransferase, *ALB* albumin

### Treatment and efficacy

**Fig. 2:**
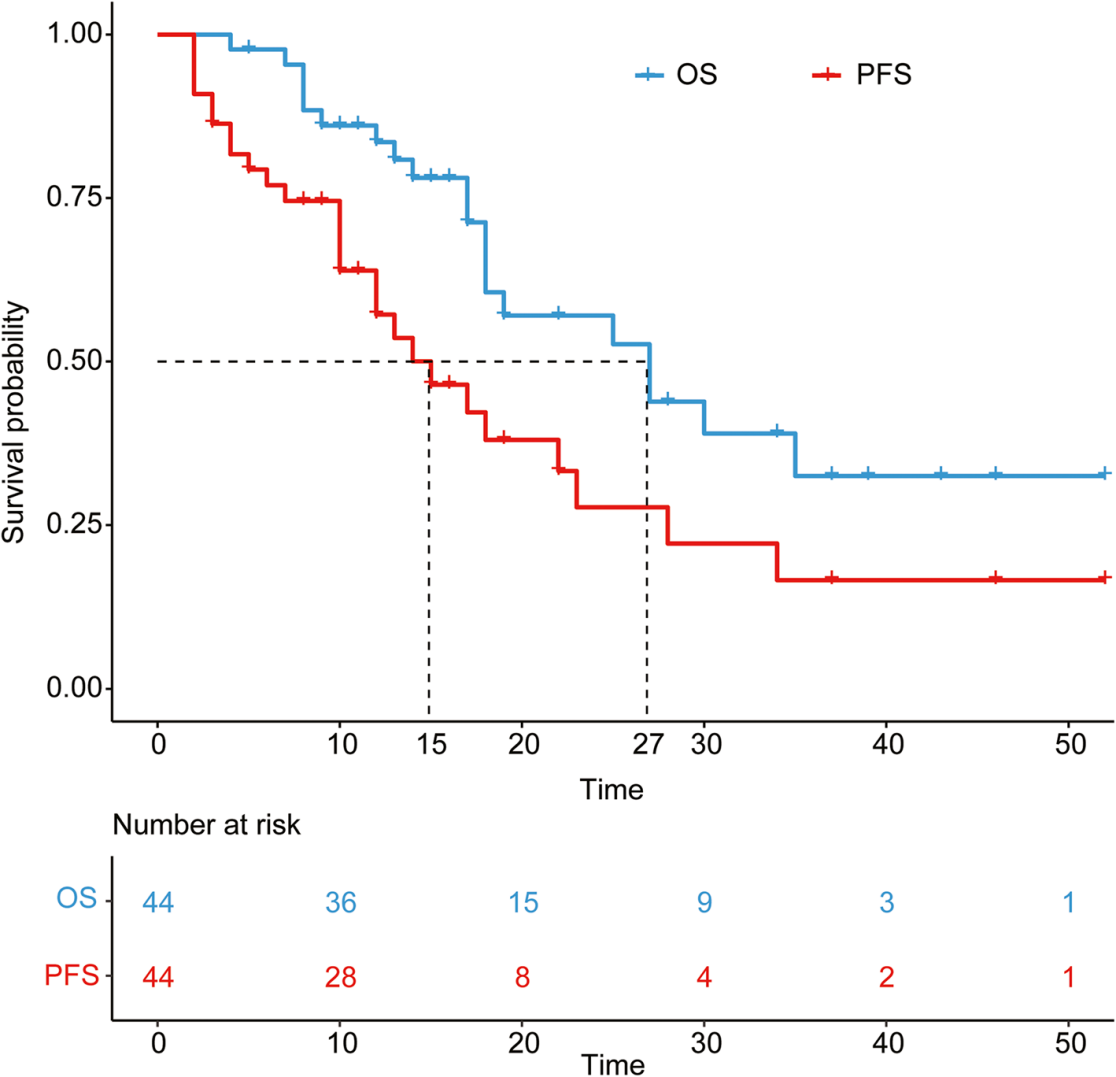
Kaplan–Meier curves of progression-free survival (PFS) and overall survival (OS), median OS was 27 months (blue), and median PFS was 15 months (red)

All patients received TKIs in combination with PD-1 inhibitors therapy, including 15 patients who received TACE, and three patients who received TACE in combination with RFA treatment (Table 2). During the follow-up period, 21 of 44 patients died of tumor progression. The assessment of treatment efficacy data is shown in Table 3. Six patients had CR (13.6%), and 12 patients (27.3%) had PR, two of whom underwent liver transplantation and secondary liver resection, respectively. SD was achieved in 10 patients (22.7%). PD was observed in 16 patients (36.4%), of which 9 patients died by the last follow-up. The ORR was 40.9%, whereas DCR was 63.6% according to the mRECIST. Median overall survival was 27.0 months (95% CI 21.2, 32.8) with a 1-year OS rate of 83.6% (95% CI 77.9%, 89.3%). Median PFS was 15.0 months (95.0% CI 12.1, 17.9), with a 1-year PFS rate of 77.0% (95% CI: 70.6%, 83.4%) (Fig. 2). The media follow-up time was 17 months after treatment.

**Table 2:** Summary of treatment

Patient no.	TKIs + ICIs	TACE/RFA
1–21	Len + Sin	~
22–31	Len + Sin	TACE
32	Len + Sin	TACE + RFA
33–34	Len + Pem	~
35–36	Len + Pem	TACE
37	Len + Niv	~
38–39	Len + Niv	TACE
40–41	Len + Niv	TACE + RFA
42	Apa + Pem	~
43	Apa + Sin	~
44	Apa + Sin	TACE

*TKIs* tyrosine kinase inhibitors, *Len* lenvatinib, *Apa* apatinib, *Sin* sintilimab, *Pem* pembrolizumab, *Niv* nivolumab, *TACE* trans-arterial chemoembolization, *RFA* radiofrequency ablation, *~* none

**Table 3:** Summary of outcomes according to mRECIST criteria

Main outcome	*N* (%)
Best tumor response	
Complete response (CR)	6 (13.6%)
Partial response (PR)	12 (27.3%)
Stable disease (SD)	10 (22.7%)
Progressive disease (PD)	16 (36.4%)
DCR	21 (63.6%)
ORR	18 (40.9%)
Median OS (months)	27.0 ± 5.8
Median PFS (months)	15.0 ± 2.9
1-year OS rate	83.6% ± 5.7%
2-year OS rate	71.3% ± 7.5%
3-year OS rate	39.0% ± 9.6%
1-year PFS rate	77.0% ± 6.4%
2-year PFS rate	63.9% ± 7.5%
3-year PFS rate	38.0% ± 8.9%

*DCR* disease control rate, *ORR* objective response rate, *OS* overall survival, *PFS* progression-free survival

### Treatment-related adverse events

The most common treatment-related adverse events (TRAEs) were elevated alanine aminotransferase (ALT) or aspartate aminotransferase (AST) (16 of 44, 36.4%), followed by diarrhea (13 of 44, 29.5%), post-embolization syndrome (PES) (13 of 18, 72.2%), skin reactions (12 of 44, 27.3%), and hypertension (10 of 44, 22.7%) (Table 4). Grade 3 TRAEs in four patients (9.1%) with the main symptoms and presentations being skin reaction (2.3%), diarrhea (6.8%), hypertension (4.5%), fatigue (2.3%), increased ALT or AST (2.3%), and hyperbilirubinemia (2.3%). There were no treatment-related deaths.

**Table 4:** Treatment-related adverse events (TRAEs)

Adverse events	Any grade, *n* (%)	Grade 3, *n* (%)
Increased ALT or AST	16 (36.4%)	1 (2.3%)
Diarrhoea	13 (29.5%)	3 (6.8%)
Post-embolization syndrome (PES)	13 (72.2%)	0
Hand-foot skin reactions	12 (27.3%)	1 (2.3%)
Hypertension	10 (22.7%)	2 (4.5%)
Fatigue	7 (15.9%)	1 (2.3%)
Arthrodynia	3 (6.8%)	0
Decreased appetite	3 (6.8%)	0
Hypothyroidism	3 (6.8%)	0
Oral mucositis	2 (4.5%)	0
Pruritus	2 (4.5%)	0
Nausea	2 (4.5%)	0
Hyperbilirubinemia	1 (2.3%)	1 (2.3%)

*ALT* alanine aminotransferase, *AST* aspartate aminotransferase, *TRAEs* treatment-related adverse events

### Presentation of two patients who underwent repeat surgery after PD-1 inhibitors in combination with TKI therapy

**Fig. 3:**
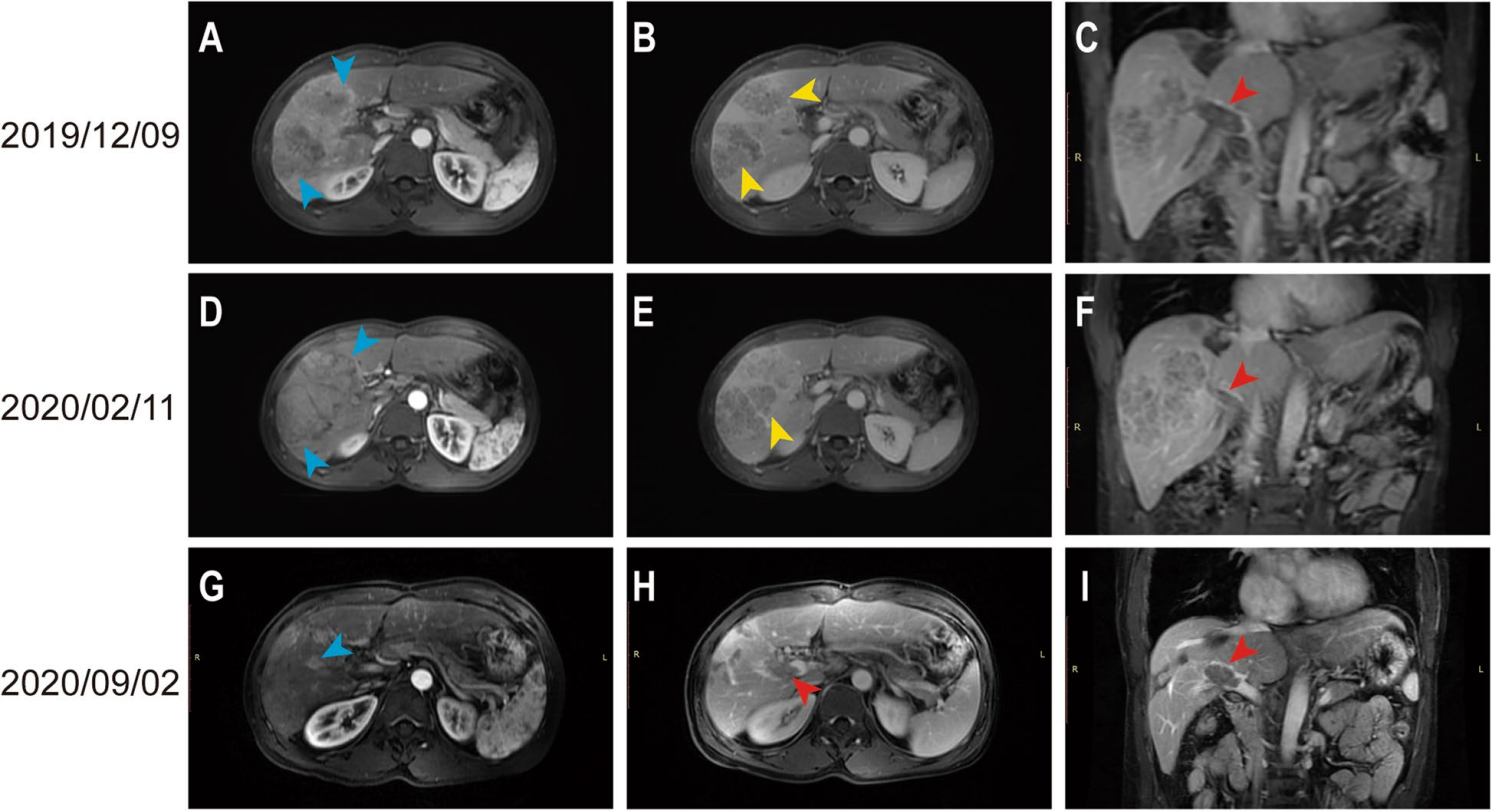
MRI examination of patient A during the combined treatment. **A**–**C** Before administering the combination therapy. **D**–**F** After 9 weeks of apatinib in combination with sintilimab therapy. **G**–**I** After 7 months of lenvatinib in combination with sintilimab therapy. The blue arrows indicate the position of lesions at the early arterial enhancement stage. The yellow arrows indicate the position of lesions at the portal vein stage. The red arrows indicate the position of PVTT

**Fig. 4:**
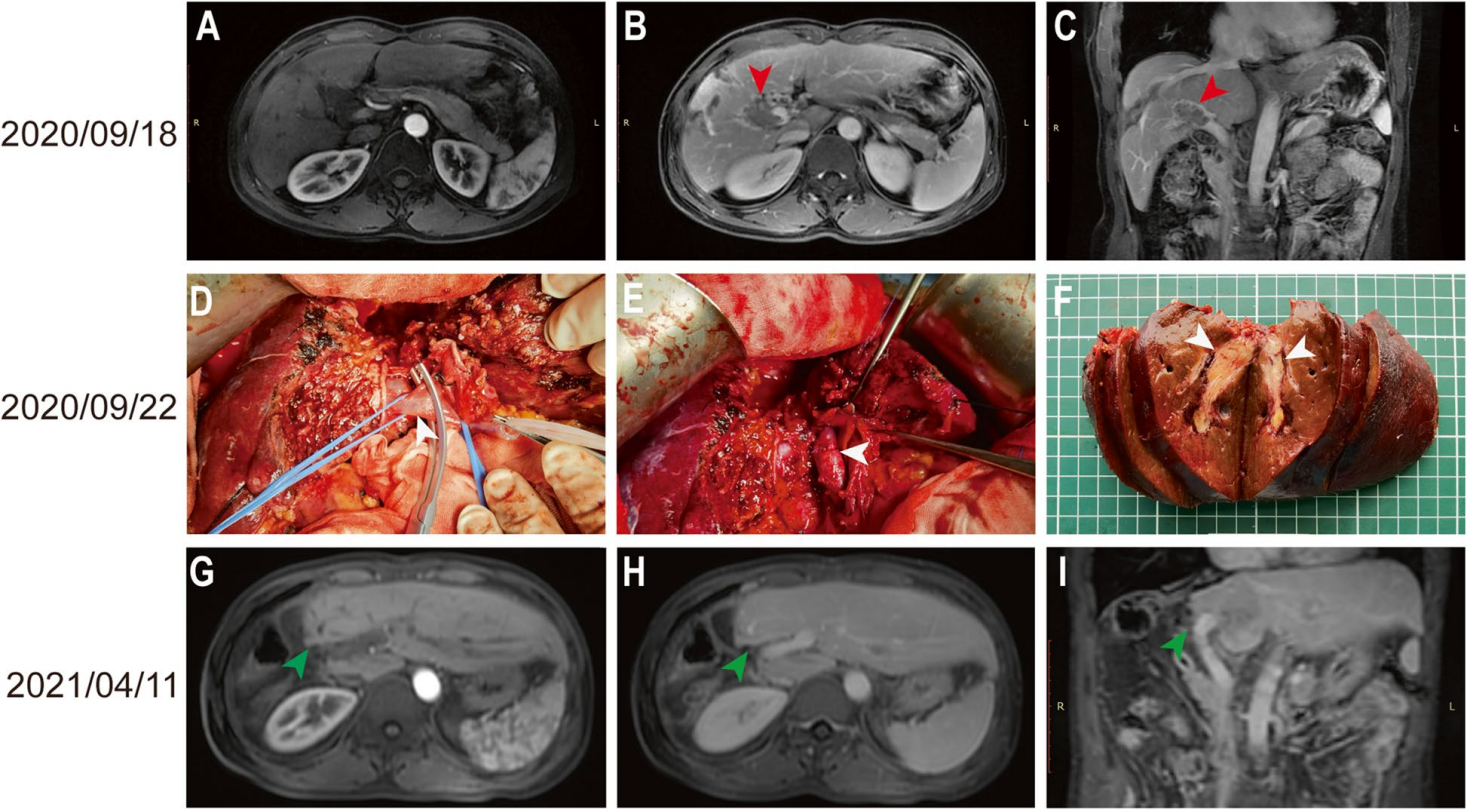
Surgical images and the MRI re-examination of patient A. **A**–**C** Pre-operative MRI images. The red arrows indicate the position of PVTT. **D** The white arrow shows the main trunk of the right branch of the portal vein. **E** The white arrow shows the sutured portal vein right branch after tumor embolectomy. **F** Specimen of the liver tissue after surgery, the white arrows show the location of the tumor. **G**–**I** MRI re-examination after surgery. The green arrows indicate the liver resection margin

A 47-year-old male patient (described as patient A) underwent right hepatic tumor resection on September 1, 2019, for primary liver cancer. The postoperative pathological examination was suggestive of a moderately and poorly differentiated hepatocellular carcinoma. The tumor was 12×7.4×7 cm in size, with intravascular tumor thrombus (> 5) around the tumor. No tumor was found in the liver margin, and the pathological stage was BCLC-A. The patient received an MRI re-examination on December 9, 2019, suggesting a new lesion in the liver with PVTT (Vp3) (Fig. 3A–C). After a comprehensive evaluation, the patient received apatinib in combination with sintilimab therapy for financial reasons firstly. After 9 weeks of therapy, the MRI re-examination suggested no significant change in tumor and PVTT (Fig. 3D–F). For a better outcome, the patient subsequently received lenvatinib in combination with sintilimab therapy. After 7 months of therapy, the MRI re-examination suggested a significant reduction in tumor diameter, and no significant changes in PVTT (Fig. 3G–I). The efficacy could be assessed as PR according to mRECIST criteria, and no significant adverse effects were observed during the treatment. After a preoperative evaluation (Fig. 4A–C), the patient underwent a right hemihepatectomy, cholecystectomy, and portal vein right branch tumor embolectomy on September 22, 2020 (Fig. 4D–F). The postoperative pathological examination revealed a localized poorly differentiated HCC (the proportion of residual tumor cells was about 20%). The total size of the tumor area was approximately 5×3×3 cm. A small amount of regressed cancerous tissue was seen in the portal vein (the proportion of residual tumor cells was about 20%). No tumor was found in the liver margin, and the pathological stage was BCLC-C. The patient recovered well and continued to receive lenvatinib in combination with sintilimab therapy 1 month after surgery. The MRI re-examination on April 11, 2021, showed no recurrence in the coelom (Fig. 4G–I). The patient has achieved survival of 34 months after combined treatment and recurrence-free survival of 25 months after repeat hepatectomy, as of November 2022.

**Fig. 5:**
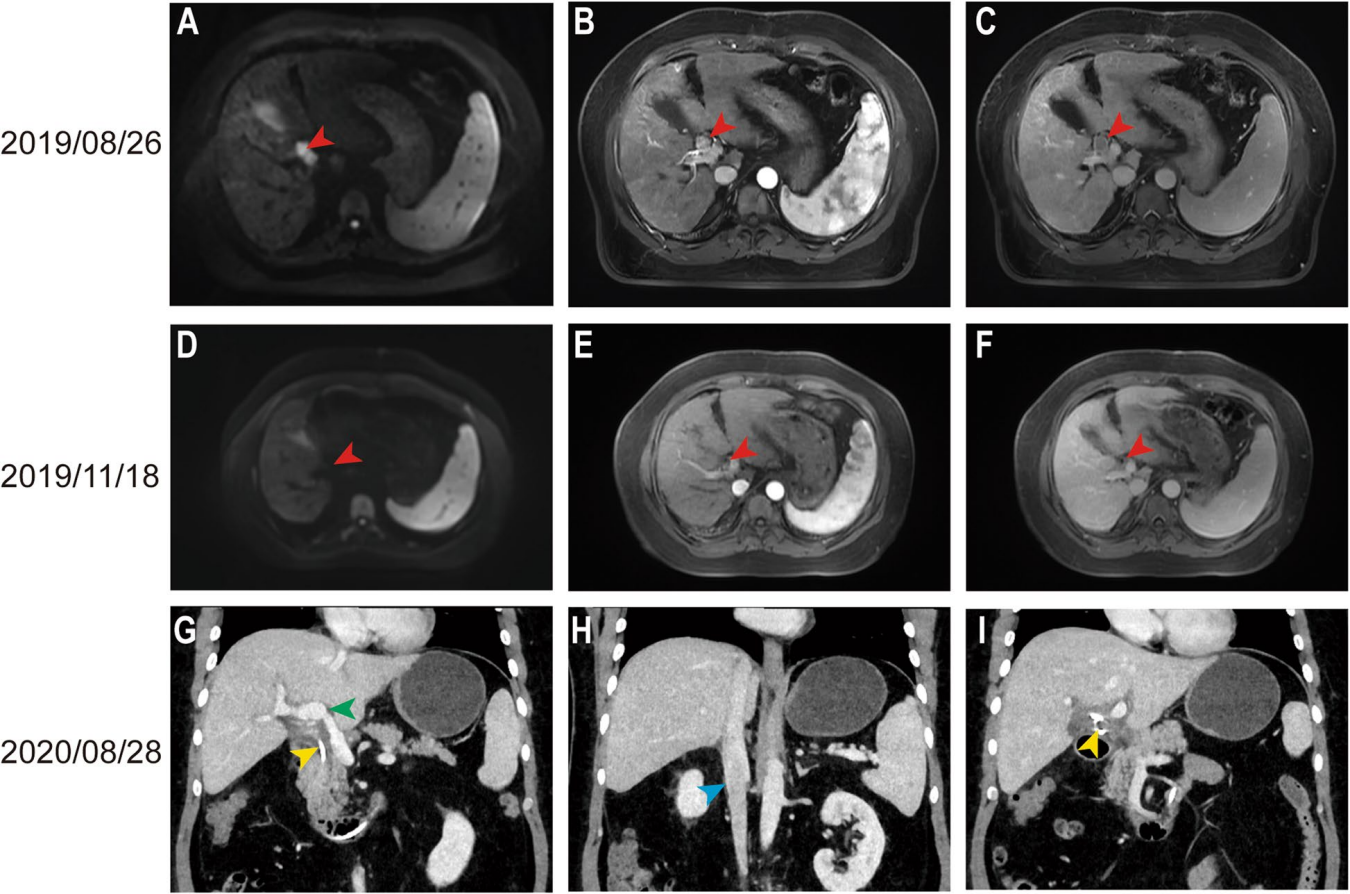
Imaging of patient B before and after treatment. **A**–**C** MRI findings at the time of recurrence. The red arrows indicate the lesion. **D**–**F** Results of MRI re-examination after 12 weeks of combined medication. The red arrow indicates the original lesion site. **G**–**I** CT scan at 6 months after liver transplantation. The green arrow indicates the portal vein, the yellow arrow indicates the bile duct, and the blue arrow indicates the inferior vena cava

Similarly, another 40-year-old male patient (described as patient B) underwent radical hepatectomy on April 28, 2019. The postoperative pathological examination was suggestive of a moderately and poorly differentiated hepatocellular carcinoma. The tumor was 2×1.8×1.2 cm in size, and no tumor was found in the liver margin. This patient on August 26, 2019, had a re-examination MRI suggesting intrahepatic recurrent lesions with hilar lymph node metastasis (Fig. 5A–C). After 12 weeks of treatment with lenvatinib combined with pembrolizumab, MRI re-examination showed significant regression of the hilar lymph nodes and a significant weakening of the arterial enhancement phase (Fig. 5D–F). The treatment efficacy can be assessed as CR according to the mRECIST criteria. The patient subsequently underwent a liver transplant on February 27, 2020, and postoperative pathology revealed no surviving carcinoma tissue in the lymph nodes. Six months after surgery, the abdominal computed tomography (CT) scan showed normal portal veins, hepatic veins, and bile ducts, with no recurrent lesions (Fig. 5G–I). The patient’s survival had reached 37 months after combined treatment, and the recurrence-free survival after liver transplantation had reached 32 months, as of November 2022.

## Discussion

Tumor recurrence is a determining factor in the OS of patients with HCC. Therefore, managing the recurrence of HCC after hepatectomy is critical to achieving long-term survival. Treatment options for unresectable recurrent HCC are limited and effective treatments need to be explored. Some studies have shown that differences in treatment are associated with survival in patients with recurrent HCC [4, 22]. In a retrospective study, 62 recurrent HCC patients with up to three tumors in the liver (each 4 cm in diameter or smaller) who were not suitable for transplantation had a median survival time of 27 months after receiving ablation; 83 patients who were not suitable for ablation had a median survival time of 19 months after receiving TACE; 44 patients who were not suitable for the above treatment options had a median survival time of 8 months after receiving systemic therapy (mainly sorafenib) [4]. Moreover, a multicenter retrospective study showed that the median survival time for two groups of patients with advanced recurrent HCC after being treated with sorafenib in combination with TACE-RFA (*n*=106) and sorafenib alone (*n*=101) was 14 months and 9 months, respectively [23]. Our study investigated the efficacy and safety of treatment based on TKIs in combination with PD-1 inhibitors for unresectable recurrent hepatocellular carcinoma. This was the first study focusing on the effect of this combination therapy for treating unresectable recurrent HCC.

Anti-VEGF therapies can reduce immunosuppression of the tumor immune microenvironment, promote tumor T cell infiltration, and may enhance the efficacy of anti-PD -1/PD-L1 [24]. Recent progress in advanced HCC has confirmed the theory and established the standard for the first-line treatment of advanced HCC. The IMbrave150 clinical trial showed that patients with unresectable HCC treated with atezolizumab-bevacizumab had a 1-year survival rate of 67.2%, a median PFS of 6.8 months, and an ORR of 33.2% (mRECIST) [13]. The combination of TKIs and immune checkpoint inhibitors has been tested in phase I trials, and some combination therapies have entered phase III trials. These TKIs have different target kinase profiles, but they all inhibit VEGFR. Lenvatinib in combination with pembrolizumab showed strong anti-tumor activity in patients with unresectable HCC with a 46% ORR (mRECIST by independent imaging review), a median PFS of 9.7 months, a median OS of 20.4 months, TRAEs greater than grade 3 occurred in 67% of patients [14]. In a retrospective clinical study, drug-eluting beads-transarterial chemoembolization (DEB-TACE) in combination with oxaliplatin-eluting calcium spheres microspheres for unresectable or recurrent HCC had a median OS of 18.8 months, with ORR and DCR of 52.4% and 95.2%, 64.7%, and 76.5%, 54.5%, and 63.3% at 1, 3, and 6 months, respectively [25]. A single-arm retrospective clinical study showed an ORR of 76.7% (23/30) and a DCR of 96.7% (29/30) (mRECIST) in patients with unresectable HCC treated with TACE-lenvatinib sequential therapy, with a median PFS of 6.1 months and a median OS of 20.7 months and acceptable adverse effects [26]. Our study shows that patients with unresectable recurrent HCC after radical surgery who have not previously received systemic therapy can also achieve long-term survival from this combination regimen, and TRAEs are manageable.

For primary HCC with macrovascular invasion and extrahepatic metastases, the American Association for the Study of Liver Diseases (AASLD) [16] and the European Association for the Study of the Liver (EASL) [27] guidelines do not recommend surgery. However, with the gradual increase in the number of studies using immune checkpoint inhibitors in combination with TKIs for the down-stage conversion of advanced HCC, some patients with unresectable HCC have been converted to resectable HCC patients who subsequently underwent surgery and eventually achieved long-term survival [28–31]. Furthermore, the efficacy of TKIs in combination with PD-1 inhibitors in patients with early recurrence of hepatocellular carcinoma after surgery has been reported in the literature [32]. In this study, the safety and efficacy of conversion therapy for unresectable recurrent HCC have been initially validated. We have also successfully applied this conversion therapy to two patients with unresectable recurrent HCC, neither of whom had seen a recurrence, as of November 2022. However, in the present study, we should still acknowledge some limitations. First, the study is a small retrospective study and may be biased. Second, the HCC patients enrolled in this study were using two different TKIs and three different PD-1 inhibitors in a complex combination. This inconsistency in medication usage may prevent studies from drawing definitive conclusions. We look forward to future prospective, large sample-size cohort studies to validate the effectiveness of this treatment option.

## Conclusion

We explored for the first time that based on the combination of TKIs with PD-1 inhibitors was safe and effective in the treatment of unresectable recurrent HCC. This study provides an option for patients with unresectable recurrent HCC.

## Data Availability

The raw data supporting the conclusions of this article is included in the article. Further inquiries can be directed to the corresponding author.
